# Vegetation degradation dominates over elevation in structuring fungal communities in alpine meadows

**DOI:** 10.3389/fmicb.2025.1596407

**Published:** 2025-07-01

**Authors:** Ni Zhu, Kexin Li, Junmei Gao, Binmeng Wei, Lirong Zhao, Lin Liu, Suyuan Jia, Laiting Zhang, Tengqi Xu, Shixiong Li, Yanlong Wang, Xiaoli Wang, Bing Liu, Yu Liu

**Affiliations:** ^1^State Key Laboratory of Soil Erosion and Dryland Farming on the Loess Plateau, Institute of Soil and Water Conservation, Northwest A&F University, Yangling, China; ^2^Key Laboratory of Degraded and Unused Land Consolidation Engineering, The Ministry of Natural Resources, Xi’an, China; ^3^Shaanxi Key Laboratory of Qinling Ecological Intelligent Monitoring and Protection, School of Ecology and Environment, Northwestern Polytechnical University, Xi’an, China; ^4^Key Laboratory of the Alpine Grassland Ecology in Three Rivers Region (Qinghai University), Ministry of Education, Xining, China; ^5^College of Life and Environmental Science, Wenzhou University, Wenzhou, China

**Keywords:** grassland degradation, elevation, fungal diversity, co-occurrence network, alpine meadow

## Abstract

**Introduction:**

Alpine meadows provide a critical natural laboratory for investigating interactions between ecosystem degradation and biogeochemical processes across elevational gradients.

**Methods:**

This study examines how degradation states and elevation (3,700 m vs. 4,300 m) influence soil fungal community composition, diversity, and network architecture in Qinghai-Tibetan Plateau grasslands. Through comparative analysis of degraded and intact meadows, we reveal fundamental shifts in belowground ecology driven by environmental change.

**Results:**

Key environmental parameters showed differential responses: soil organic matter (SOM) decreased significantly with degradation, while soil water content exhibited elevation-dependent patterns (*p* < 0.05). High-throughput sequencing identified Ascomycota, Mortierellomycota, and Basidiomycota as dominant phyla across all samples. Redundancy analysis (RDA) analysis demonstrated that edaphic factors explained 71.3% of fungal community variation, with SOM emerging as the principal driver (*p* = 0.001). Interestingly, meadow degradation led to an increase in fungal species diversity, thereby simplifying network complexity. Fungal communities show greater sensitivity to degradation than elevational gradients.

**Discussion:**

Our results provide a mechanistic framework for predicting fungal community responses to environmental change, with implications for alpine ecosystem management. Future restoration efforts should prioritize SOM conservation and monitor network properties as early warning indicators of ecosystem degradation.

## 1 Introduction

Soil fungal communities orchestrate critical ecosystem processes through decomposition, symbiosis, and pathogenicity, yet their responses to interacting environmental stressors remain poorly resolved in alpine systems (Tedersoo et al., 2014; Peay et al., 2016; Mayer et al., 2021). Soil fungi have been demonstrated exceptional environmental sensitivity, with their taxonomic plasticity and functional redundancy enabling rapid adaptation to shifting conditions (Miyamoto et al., 2014; Peay et al., 2016). Mountain slopes, characterized by multiple changing variables such as climate and soil, serve as complex environmental gradients for studying the impacts of climate change. Montane ecosystems provide a natural laboratory to investigate these dynamics, where compressed environmental gradients create microcosms of climate change impacts (Xu et al., 2014). Changes in temperature and moisture along these gradients can lead to shifts in the structure (Van Nuland et al., 2017), diversity (Yang et al., 2014), and ecological functions (Jarvis et al., 2015) of soil microbial communities. Specifically, soil fungal richness often declines at higher elevations or peaks at mid-elevations, a pattern consistently observed in both fungal and other microbial communities. This decline in richness at higher elevations is typically attributed to harsher climatic conditions, reduced energy availability, and poorer soil quality (Yang et al., 2022). Additionally, the loss of rare species under environmental stress at high elevations further contributes to this trend (Barbi et al., 2025). Conversely, the mid-domain effect may explain the mid-elevation peak in species richness (Truong et al., 2019).

The Qinghai-Tibetan Plateau’s (QTP) extreme elevational gradients create a natural experiment for disentangling fungal community responses to environmental filters, yet emerging patterns remain enigmatic (Breidenbach et al., 2022; Chen et al., 2022; Kang et al., 2022). Fungal diversity along elevational gradients displays various trends, including unchanging, decreasing, increasing, or unimodal patterns (Jarvis et al., 2015; Siles and Margesin, 2016; Ji et al., 2022; Li et al., 2022). While fungal α-diversity typically declines with elevation in temperate systems (Jarvis et al., 2015). Previous studies reveal taxon-specific responses defying universal rules, ascomycota exhibit elevational indifference in southeastern subregions (Liu D. et al., 2018; Liu Y. H. et al., 2018), whereas Himalayan slopes show U-shaped diversity patterns linked to microclimatic thresholds (Yang et al., 2022). Paradoxically, metagenomic analyses report elevational increases in functional gene abundance (Hussain et al., 2022), contrasting with observed α-diversity declines (Wang Q. et al., 2022; Wang X. S. et al., 2022)—a disparity suggesting methodological biases (e.g., primer selection) or scale-dependent responses. Therefore, understanding and predicting the diversity patterns of fungal communities in alpine meadows is of paramount importance.

Grassland degradation triggers cascading effects on belowground ecosystems, with plant biomass reduction and root exudate depletion fundamentally restructuring fungal niches (Wu et al., 2014). Although numerous studies have examined the effects of grassland degradation on soil microbial communities (Abdalla et al., 2018), recent findings suggest that while the α-diversity of bacterial and fungal communities may remain relatively stable across different degradation stages, their composition undergoes substantial shifts (Zhou et al., 2019). Notably, fungi appear to be particularly sensitive to degradation, with their relative abundance at the phylum level declining as degradation progresses (Ma et al., 2024). However, in lightly degraded alpine meadows on the QTP, fungal community species richness has been found to increase significantly compared to non-degraded areas (Zhao et al., 2021). Degradation also alters soil physicochemical properties, which in turn affects soil fungal communities. Soil pH and sand content typically increase, while moisture, organic matter, clay, and silt content decrease (Li et al., 2023; Liu D. et al., 2018; Liu Y. H. et al., 2018). These changes can lead to declines in soil microbial biomass and significant shifts in fungal β-diversity (Cao et al., 2011; Luo et al., 2014). Understanding the complex interactions between microbial activity and soil properties in degraded grasslands is crucial for developing effective restoration strategies.

In natural ecosystems, microbial species form complex ecological networks through interconnections (Faust and Raes, 2012; Chen et al., 2020). Co-occurrence networks have been used to study microbial taxon interactions, where nodes represent microbial species and edges indicate their correlations (Ma et al., 2020; Yu et al., 2024). Both grassland degradation and elevation changes can significantly impact the structure of soil microbial networks, potentially altering their stability and function (Wu et al., 2021; Li et al., 2022). Grassland degradation affects soil properties, which in turn influences microbial co-occurrence patterns. As degradation progresses, microbial co-occurrence networks may become more complex, often associated with decreased nutrient availability and increased soil heterogeneity (Wardle et al., 2012). Elevational increases can lead to significant declines in topological features of fungal co-occurrence networks, such as average degree and clustering coefficient, possibly due to tighter community associations at lower elevations (Yang et al., 2021).

The QTP with its unique geography and harsh climate, presents a challenging environment for microorganisms. This fragile ecosystem is particularly sensitive to climate change and anthropogenic disturbances (Xu et al., 2014). Understanding the vertical distribution of fungal communities and the impact of vegetation degradation is crucial for grasping microbial dynamics in alpine regions. However, the interplay between vegetation degradation and elevational gradients on fungal communities remains unclear. This study investigates the relationships among vegetation, soil properties, and fungal communities along an elevational gradient, exploring their influences on fungal community composition and structure. We posit that both vegetation and elevation significantly affect fungal communities, with vegetation degradation having a more pronounced impact than elevation (Miyamoto et al., 2014; Li et al., 2022). Our research addresses the following questions: (1) How do fungal community composition and diversity respond to elevational gradients and vegetation degradation? (2) What associations exist between soil fungal communities and soil physicochemical properties at varying elevations and levels of grassland degradation? (3) Which factor-vegetation or elevation-plays a more dominant role in shaping fungal communities in montane areas? By examining these aspects, our study aims to enhance the understanding of fungal responses to global change and contribute to the knowledge of fungal community dynamics in alpine meadows.

## 2 Materials and methods

### 2.1 Study area

The experiment was conducted in Dari County, Qinghai Province, China (32°36′–34°15′N, 98°15′–100°33′E), at an average elevation of approximately 4,200 meters. This region experiences a subhumid alpine climate characterized by a lack of distinct seasons, with alternating cold and warm periods and no frost-free days. The cold season, lasting 7 to 8 months, is marked by frequent winds and snowfall, while the warm season is short, spanning only 4 to 5 months. The average annual temperature in the study area is below −1.2°C, with the coldest and hottest monthly temperatures recorded at −12.9°C in January and 9.1°C in July, respectively (Liu et al., 2023). Annual precipitation averages between 486.9 mm and 666.5 mm, primarily occurring from May to September (Qian et al., 2024). The region features diverse soil types, including felty soils and black clay soils. Dominant vegetation types comprise alpine meadows, alpine shrubs, and swamp meadows (Liu et al., 2022), with key native plant species including *Kobresia pygmaea*, *Potentilla saundersiana*, *Polygonum viviparum*, *Potentilla fruticosa*, and *Ligularia virgaurea*.

### 2.2 Experimental design and sampling

Four study sites with varying meadow vegetation conditions (extremely degraded and non-degraded) and elevations (low 3,700 m and high 4,300 m) were randomly selected, ensuring a minimum distance of 100 meters between each site at the same elevation. The experimental treatments included: low elevation with extremely degraded grassland (Low-De), low elevation with non-degraded grassland (Low-Un), high elevation with extremely degraded grassland (High-De), and high elevation with non-degraded grassland (High-Un). Following the removal of meadow vegetation, three soil cores were randomly collected from each study site at a depth of 0–10 cm using a soil auger (5 cm diameter). The soil samples were passed through a 2 mm sieve to eliminate stones, roots, and plant residues, and then air-dried in a ventilated area until constant weight for subsequent analysis of physicochemical properties. Additionally, four surface soil cores were collected from each site and stored at −80°C for high-throughput sequencing analysis.

Soil organic matter (SOM) content was quantified using the potassium dichromate-concentrated sulfuric acid oxidation method. Soil water content (SWC) was determined using the oven-drying method. Soil particle composition (SPC) was analyzed using a Mastersizer 3000 laser particle analyzer to quantify the proportions of clay particles (0.0001–0.002 mm), silt particles (0.002–0.05 mm), and sand particles (0.05 mm–2 mm).

### 2.3 DNA extraction and high-throughput sequencing

Soil (0.5 g) was taken from each sample using a specialized kit (Qiagen DNeasy PowerSoil Kit, Venlo, Netherlands) for DNA extraction to maximize the recovery of DNA. The integrity of the extracted DNA was assessed via 1% agarose gel electrophoresis, and the DNA concentration and purity were quantified using a multi-mode microplate reader (Tecan Ltd., Switzerland). The fungal internal transcribed spacer (ITS1) region was amplified with the primers ITS5F (5′-GGAAGTAAAAGTCGTAACAAGG-3′) and ITS2R (5′-GCTGCGTTCTTCATCGATGC-3′), incorporating a barcode. A small fragment library was constructed and sequenced on the Illumina HiSeq platform using paired-end sequencing. Post-homogenization, the samples were submitted to Beijing Biomarker Technologies Co., Ltd. for amplicon sequencing.

Raw sequences were assembled and subjected to quality filtering by overlapping and merging using FLASH version 1.2.7. Chimera sequences were identified and eliminated using UCHIME version 4.2, yielding effective tags. Operational taxonomic units (OTUs) were generated by clustering tags at a 97% similarity threshold using usearch software (version 8.1.1831). Taxonomic analysis of representative fungal sequences was performed with the UNITE database. Alpha diversity indices, including ACE, Chao1, Simpson, and Shannon, were calculated using Mothur (version 1.30) to evaluate soil fungal diversity across study sites.

### 2.4 Statistical analyses

The effects of meadow vegetation, elevation, and their interactions were evaluated using two-way analysis of variance (ANOVA) in SPSS. One-way ANOVA, followed by the least significant difference (LSD) *post hoc* test, was employed to determine significant differences in SOM, SWC, SPC, and fungal alpha diversity among treatments (High-Un, High-De, Low-Un, and Low-De) for the 0–10 cm soil depth. The ternary phase diagram was constructed using the R “ggtern” package to represent soil silt, clay, and sand contents. Principal coordinates analysis (PCoA) with the Bray–Curtis distance, implemented in the R “vegan” package, was used to assess soil fungal variations across treatments. PERMANOVA, based on the Bray–Curtis distance matrix, was conducted in R (vegan package, adonis function) to test the significance of elevation and grassland on soil fungal communities. Redundancy analysis (RDA) and Pearson correlation analysis, conducted with the “vegan” and “corrplot” packages in R, respectively, were used to explore correlations between soil properties and soil fungal community composition and diversity. A robust correlation network was constructed based on a Spearman correlation coefficient of *r* > 0.7 and *p* < 0.05. Topological coefficients were calculated using the “igraph” package, and the soil fungal co-occurrence network was visualized with Gephi-0.10.1. Statistical significance was set at *p* < 0.05 for all analyses.

## 3 Results

### 3.1 Impact of elevation and grassland vegetation on soil properties

**Figure 1:**
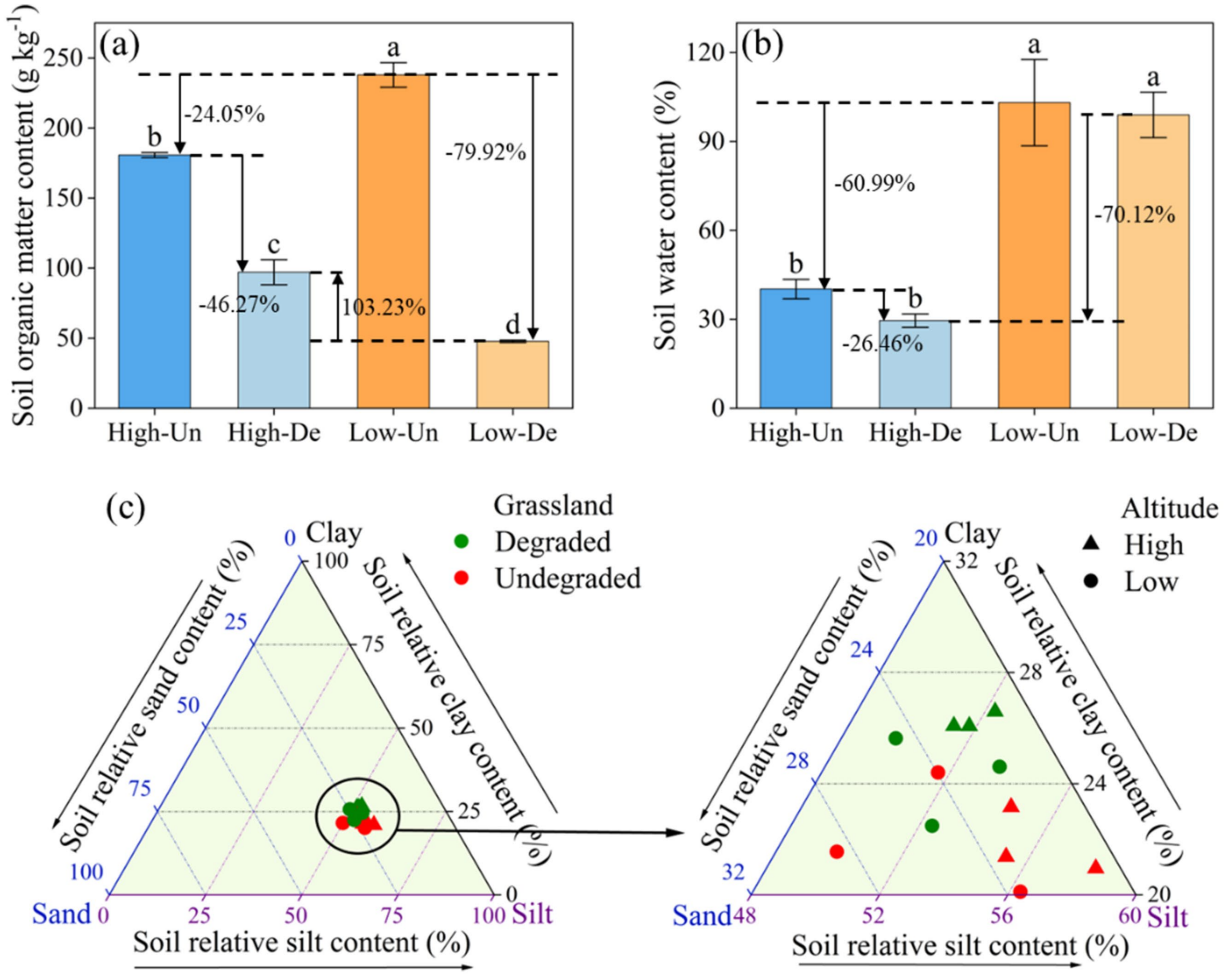
Effects of elevation and meadow vegetation on the contents of **(a)** soil organic matter (SOM), **(b)** soil moisture (SWC), and **(c)** soil particle composite in 0–10 cm. Different lowercase letters indicate significant differences among treatments at 0.05 level. Values are the mean ± standard error. High-Un, high elevation and non-degraded grassland; High-De, high elevation and degraded grassland; Low-Un, low elevation and non-degraded grassland; Low-De, low elevation and degraded grassland.

The concurrent degradation of meadow vegetation and soil had a pronounced effect on soil organic matter (SOM) content, with a significant interaction between vegetation and elevation (Table 1). SOM content was markedly higher in Low-Un meadows (237.90 g kg^−1^), with degradation leading to a 24.05 and 79.72% decrease in SOM for High-Un and Low-De meadows, respectively. The SOM content in High-De meadows decreased by 46.27% compared to High-Un but increased by 103.23% compared to Low-De (Figure 1a, *p* < 0.05). Soil moisture content in high-elevation meadows showing a 60.99 and 70.12% reduction in soil water content compared to Low-Un and Low-De, respectively (Figure 1b, *p* < 0.05). Grassland vegetation significantly influenced soil clay content, while elevation significantly affected soil sand content (Table 1). The soil particle composition, dominated by silt, was further detailed by a ternary plot (Figure 1c), with clay content being highest in High-De (26.25%) and no significant differences in silt and sand content among treatments (*p* > 0.05).

**Table 1:** Two-way analysis of variance (ANOVA) of soil properties in 0–10 cm soil depth.

Soil properties	Meadow vegetation		Elevation		Meadow vegetation * elevation	
	*F* value	*p*-value	*F* value	*p*-value	*F* value	*p*-value
SOM	466.857	**<0.001**	0.390	0.550	70.703	**<0.001**
SWC	0.760	0.409	61.049	**<0.001**	0.148	0.710
Clay	14.999	**0.005**	1.118	0.321	1.625	0.235
Silt	3.946	0.082	1.753	0.222	1.884	0.207
Sand	0.615	0.456	6.139	**0.038**	0.367	0.561

Bold letters indicated statistically significant differences (*p* < 0.05). SOM, Soil organic matter; SWC, soil water content; Clay, soil clay content; Slit, soil silt content; Sand, soil sand content.

### 3.2 Soil fungal community composition and diversity across environments

**Figure 2:**
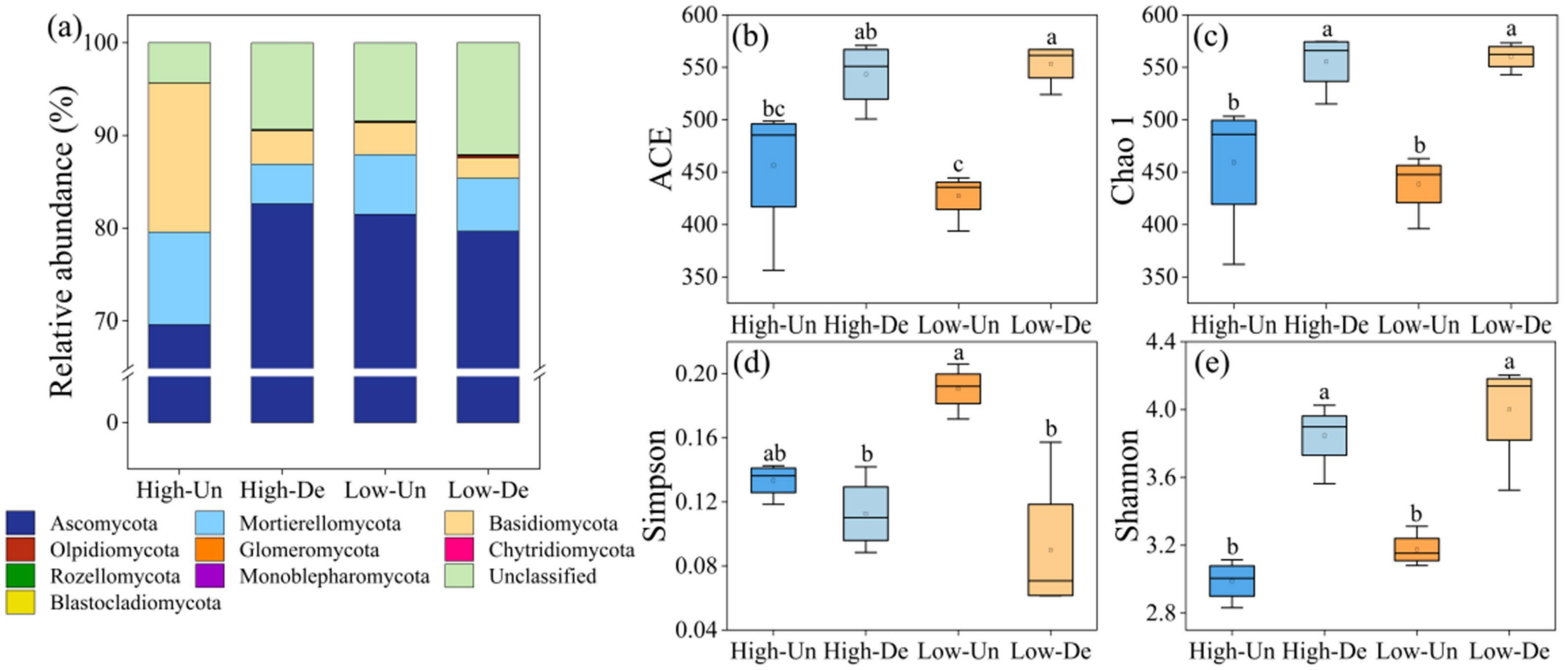
**(a)** Relative abundance of the nine most abundant fungal phyla. Alpha diversity of the soil fungal community including **(b)** ACE index. **(c)** Chao 1 index. **(d)** Simpson index. **(e)** Shannon index with different treatments. Different lowercase letters indicate significant differences among treatments at 0.05 level. High-Un, high elevation and non-degraded grassland; High-De, high elevation and degraded grassland; Low-Un, low elevation and non-degraded grassland; Low-De, low elevation and degraded grassland.

Ascomycota, Mortierellomycota, and Basidiomycota were the predominant fungal phyla, with Ascomycota accounting for over 69.58% of the relative abundance across all treatments (Figure 2a). Mortierellomycota and Basidiomycota were more abundant in non-degraded grasslands at the same elevation, and their relative abundances decreased with increasing elevation in non-degraded grasslands, while Ascomycota increased. In contrast, degraded grasslands at lower elevations showed decreased relative abundances of Ascomycota and Basidiomycota but increased Mortierellomycota (Figure 2a).

Alpha diversity indices-ACE, Chao1, Simpson, and Shannon-revealed that grassland degradation significantly affected fungal alpha diversity, and the interaction between grassland and elevation significantly influenced the Simpson index (Table 2). The ACE, Chao1, and Shannon indices were significantly higher in degraded grasslands at the same elevation (Figures 2b–d), with no significant differences observed between elevations within the same degradation level. The Low-De treatment exhibited the highest soil fungal species richness, as indicated by the ACE and Chao1 indices, and the greatest species diversity, as shown by the lowest Simpson index and highest Shannon index (Figures 2b–d).

**Table 2:** Two-way analysis of variance (ANOVA) of soil fungal alpha diversity in different treatments.

Factor	Meadow vegetation		Elevation		Meadow vegetation * elevation	
	*F* value	*p*-value	*F* value	*p*-value	*F* value	*p*-value
ACE	28.027	**<0.001**	0.223	0.645	0.952	0.349
Chao1	30.858	**<0.001**	0.166	0.691	0.424	0.527
Simpson	20.199	**<0.001**	1.634	0.225	8.731	**0.012**
Shannon	68.355	**<0.001**	2.794	0.120	0.023	0.882

Bold letters indicated statistically significant differences (*p* < 0.05).

**Figure 3:**
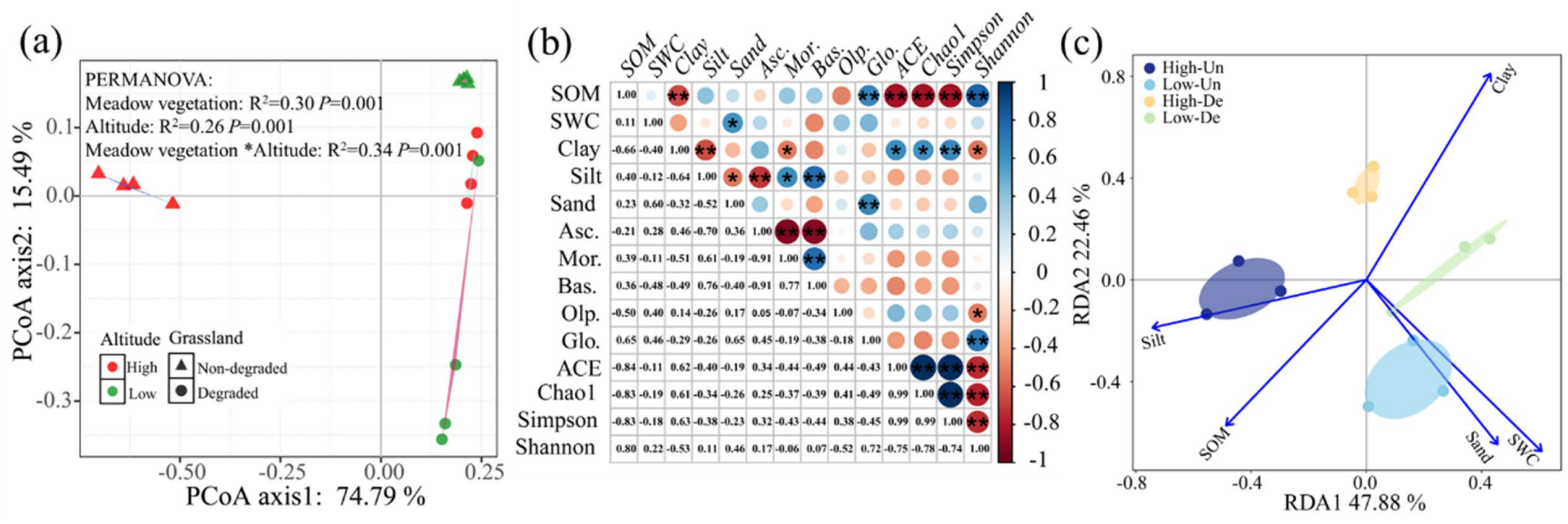
**(a)** Principal coordinate analysis (PCoA) of soil fungal community. Beta diversity was analyzed by PCoA based on Bray–Curtis distance at the operational taxonomic unit (OTU) level. **(b)** Correlations between fungal community and soil properties (^*^p < 0.05 and ^**^p < 0.01). **(c)** Redundancy analysis (RDA) revealed linkages between fungal community (symbols) and soil properties (arrows). The value of the axis is the variance percentage explained for the axis. The analysis was performed at the operational taxonomic unit (OTU) level. SOM, soil organic matter; SWC, soil water content; Clay, soil clay content; Silt, soil silt content; Sand, soil sand content; Asc., Ascomycota; Mor., Mortierellomycota; Bas., Basidiomycota; Olp., Olpidiomycota; Glo., Glomeromycota; High-Un, high elevation and non-degraded grassland; High-De, high elevation and degraded grassland; Low-Un, low elevation and non-degraded grassland; Low-De, low elevation and degraded grassland.

PCoA analysis indicated that elevation, grassland, and their interaction significantly influenced fungal community composition, explaining 90.28% of the variation, with the first and second axes accounting for 74.79 and 15.49%, respectively (Figure 3a). The fungal communities in High-De and Low-De treatments were more similar, while the greatest differences were observed between High-Un and Low-Un treatments (Figure 3a and Table 3).

**Table 3:** Adonis analysis of between-group variations in soil fungal communities.

Pairs	*R* ^2^	*p*-value	*p*-adjusted
High-Un vs. Low-Un	0.96	0.035	0.043
High-Un vs. High-De	0.94	0.030	0.043
High-Un vs. Low-De	0.88	0.043	0.043
Low-Un vs. High-De	0.69	0.031	0.043
Low-Un vs. Low-De	0.66	0.020	0.043
High-De vs. Low-De	0.55	0.036	0.043

High-Un, high elevation and non-degraded meadow vegetation; High-De, high elevation and degraded meadow vegetation; Low-Un, low elevation and non-degraded meadow vegetation; Low-De, low elevation and degraded meadow vegetation.

### 3.3 Vegetation mediates fungal communities via soil properties

Pearson correlation analysis revealed significant correlations between soil properties and soil fungal community composition and diversity (Figure 3b). SOM and soil clay content were significantly correlated with fungal alpha diversity, with Olpidiomycota negatively correlated with the Shannon index and Glomeromycota positively correlated. Silt content was significantly correlated with fungal community composition, while SOM and sand content were positively correlated with Glomeromycota, and clay content negatively correlated with Mortierellomycota. Additionally, SOM was negatively correlated with clay content, and SWC was positively correlated with soil sand content.

RDA analysis showed that soil properties explained 71.30% of the variation in fungal community composition, with 49.24 and 22.06% attributed to the first and second axes, respectively (Figure 3c). Silt and SWC were the main contributors to the first axis, while SOM, clay, and sand had stronger influences on the second axis. The Monte Carlo test confirmed significant correlations between SOM, SWC, clay, sand, and silt with fungal communities (*p* < 0.05; Table 4). Among all soil properties, Clay was the most critical factor in determining fungal community structure (*p* < 0.01).

**Table 4:** Goodness-of-fit statistics (*R*^2^) for environmental factors fitted to the constrained redundancy analysis (RDA).

Factor	RDA1	RDA2	*R* ^2^	*p*-value
SOM	−0.4426	−0.8967	0.60	**0.024**
SWC	0.6364	−0.7714	0.70	**0.005**
Clay	0.4478	0.8941	0.80	**0.003**
Sand	0.5170	−0.8560	0.53	**0.022**
Silt	−0.9510	−0.3093	0.50	**0.050**

SOM, soil organic matter; SWC, soil water content; Clay, soil clay content; Slit, soil silt content; Sand, soil sand content.

**Figure 4:**
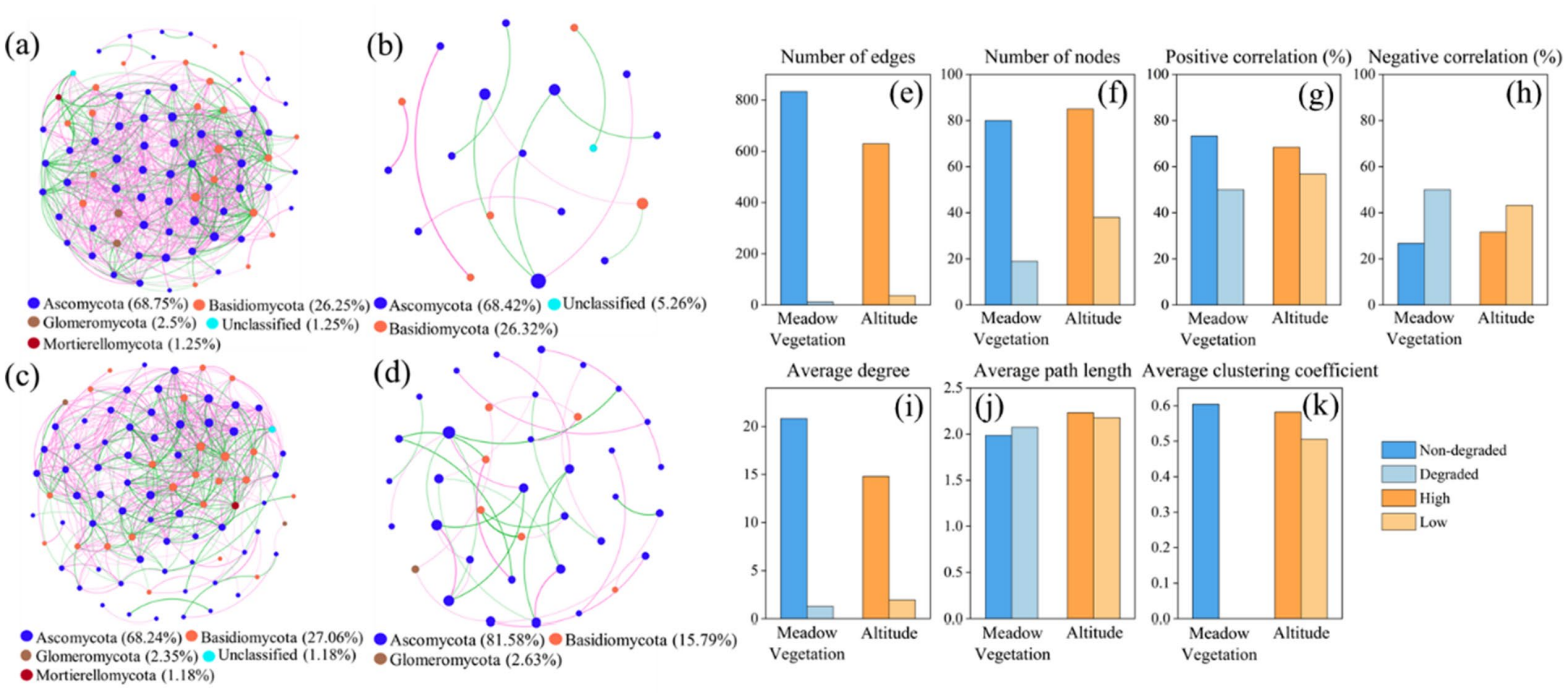
Soil fungal co-occurrence network in **(a)** Non-degraded grassland. **(b)** Degraded grassland. **(c)** High elevation. **(d)** Low elevation. Topological properties of the fungal co-occurrence network including **(e)** Number of edges. **(f)** Number of nodes. **(g)** Positive correlation (%). **(h)** Negative correlation (%). **(i)** Average degree. **(j)** Average path length. **(k)** Average clustering coefficient in meadow vegetation and elevation. The size of the points represents the abundance, the thickness of the lines represents the magnitude of the correlation coefficient, green lines represent negative correlation, and pink lines represent positive correlation.

Fungal co-occurrence networks at different elevations and degradation levels revealed that nodes were primarily affiliated with Ascomycota and Basidiomycota (Figure 4). Non-degraded grasslands and high elevations formed more complex networks with a higher proportion of positive correlations. Grassland degradation and lower elevations reduced network complexity, as evidenced by a decrease in the number of edges and nodes, average degree, and average clustering coefficient, and an increase in negative correlation. The decrease in topological features of the fungal co-occurrence network was more pronounced with vegetation changes.

## 4 Discussion

### 4.1 Variation of key soil variables with elevation and vegetation

Elevation is a pivotal ecological factor in mountain ecosystems, driving high heterogeneity in vegetation, temperature, and soil properties (Njeru et al., 2017). We observed a decline in SOM with increasing elevation in non-degraded grasslands (Figure 1a), aligning with the vertical distribution of soil organic carbon reported by Hou et al. (2021) in the central QTP. In alpine grasslands, plant-derived carbon input is a primary source of soil organic carbon (Li et al., 2017), with higher plant biomass, particularly underground, correlating with increased soil organic carbon concentrations. The higher SOM at lower elevations can be attributed to higher rainfall, favorable temperatures, and better water–heat conditions, which promote greater plant community diversity and abundance (Yang et al., 2014; Barbi et al., 2025). Consequently, high-quality and high-biomass litter contributes more nutrients to the soil, enhancing SOM accumulation.

Vegetation degradation significantly reduces plant biomass, soil aggregation, and nutrient content, exposing the soil and accelerating SOM decomposition (Breidenbach et al., 2022). However, in high-elevation areas, lower temperatures and longer soil freezing periods slow down the decomposition process. Similarly, lower temperatures and reduced vegetation coverage at high elevations increase soil water evaporation and decrease soil water-retention capacity, reducing soil moisture (*p* < 0.001, Table 1). Grassland degradation leads to a significant loss of soil fertility (Sun et al., 2024; She et al., 2022). As grassland degradation progresses, plant biomass and litter decrease, underground plant carbon input declines, and reduced vegetation cover promotes wind and water erosion, resulting in lower SOM (Chappell et al., 2013; Zhang et al., 2019). Long-term monitoring of sensitive SOM changes in response to the degradation gradient is crucial for uncovering the risk of grassland degradation in its early stages.

### 4.2 Impact of meadow degradation on soil fungal community stronger than elevation

Fungi form symbiotic relationships with plant roots to enhance nutrient utilization efficiency and participate in the decomposition of plant litter and residues (Kubartová et al., 2009). Both elevation and vegetation play significant roles in the composition of fungal communities. Ascomycota was the dominant fungal phylum in alpine meadows (Li et al., 2016), likely due to its high tolerance and turnover rates, enabling it to better withstand environmental stress and enhance its dominance in degraded grasslands (Cho et al., 2009). The relative abundance of Basidiomycota showed a declining trend during the degradation process, as it is typically found in areas of higher soil quality, and grassland degradation alters the soil environment, leading to its reduced abundance (de Araujo et al., 2017). Mortierellomycota, which reflects soil organic matter decomposition, soil fertility, and microbial community structure, plays a significant role in assessing soil health and quality due to its diversity and abundance (Ning et al., 2022). These abundant fungal phyla showed higher diversity in vegetation rather than elevational gradients (Figure 3a), supported by fungal α-diversity (Figures 2b–e). These results indicate that vegetation has a greater effect on fungal communities than elevation, attributed to changes in soil properties.

Degraded grasslands showed significantly higher soil fungal diversity compared to non-degraded grasslands (Figures 2b–e), consistent with previous research on soil fungal communities in the QTP (Li et al., 2016; Jiang et al., 2024; Wang Q. et al., 2022; Wang X. S. et al., 2022). Vegetation degradation significantly alters the physicochemical properties of soil and further changes soil fungal communities (Wang et al., 2021). Redundancy analysis showed that the dominant drivers of soil fungal community structure were soil organic matter, soil water content, and soil particle composition. Grassland degradation reduces soil nutrients and restricts plant growth, leading to decreased soil organic carbon accumulation, which allows fungal communities to dominate the microbial community (Ao et al., 2024). Moreover, grassland degradation reduces plant biomass and diversity, leading to the deterioration of soil quality and plant health, weakening plant resistance and thereby increasing the abundance of pathogenic fungi, which could increase the potential risk to the health of alpine plant-soil ecosystems (Li et al., 2016; Bennett and Klironomos, 2019). Additionally, the destruction of the turf layer accelerates soil erosion by water and wind, promoting the dispersal of pathogenic fungi and increasing soil fungal diversity (Che et al., 2019). Previous studies also suggested that increased fungal diversity due to degradation was highly related to microbial homogenization rather than species richness (Wu et al., 2024). The homogenization of microbial communities reduces the complexity of co-occurring networks. Our results confirm that grassland degradation and decreasing elevation reduced the complexity of soil fungal networks, mainly by lowering network topology parameters such as the number of edges, nodes, average degree, and average clustering coefficient (Figures 4e–k).

Co-occurring network complexity is more sensitive to degradation than elevation. As grassland degradation increases, soil moisture content, soil organic matter, total nitrogen, and total phosphorus significantly decrease, while the composition and diversity of soil fungal communities change, and the number of nodes and the average clustering coefficient in the fungal co-occurrence network significantly decrease, leading to a more fragmented network structure that tends toward homogenization and reduced stability. Grassland degradation reduces clustering within the fungal community co-occurrence network, weakening interactions and leading to a more dispersed and homogeneous survival strategy in soil fungi, which indirectly decreases the stability of the fungal co-occurrence network (Faust and Raes, 2012; Coyte et al., 2015; Chen et al., 2021). These changes in fungal communities mark an irreversible course of grassland degradation in the short term (Breidenbach et al., 2022). However, non-degraded grasslands had a larger network size and more complex structure (Figure 4a), suggesting stronger resistance to environmental disturbances (Santolini and Barabási, 2018; de Vries et al., 2018). The increase in elevation leads to changes in climate and plant communities, which alters soil moisture, temperature, pH, and nutrient availability (Jarvis et al., 2015; Nottingham et al., 2018; Yang et al., 2022). These changes directly impact the structure of soil fungal communities (Miyamoto et al., 2014; Zhang et al., 2024). Studies have shown that *Dasiphora fruticosa* increased the heterogeneity of microbial communities and microenvironments, thereby altering the impact of elevation on microbial diversity and function (Wang Q. et al., 2022; Wang X. S. et al., 2022). Although environmental changes brought about by elevation have a significant effect on soil fungal communities, this process is reversible or even compensated for by the restoration of vegetation. When environmental conditions (plant community composition and climate) change, soil properties (such as pH and SOC) are also altered, leading to the recovery or adjustment of fungal communities. Overall, grassland degradation had stronger effects on the diversity of soil fungal communities than elevation, emphasizing the vulnerability and sensitivity of soil fungi to grassland degradation.

## 5 Conclusion

Our study provides a systematic insight into the dynamics of soil fungal communities in alpine meadows, highlighting the effects of degradation and elevational changes. We found that the degradation of meadow vegetation significantly decreased the soil organic matter content, while elevation exerted a substantial influence on soil water content in these ecosystems. Notably, soil fungal communities were more sensitive to the impacts of grassland degradation than to changes in elevation. Degradation of meadows led to an increase in fungal species diversity, however, which in turn reduced the complexity of the fungal co-occurrence network. These findings suggest that soil fungal community diversity and network complexity can serve as robust indicators of grassland health and restoration status. They hold practical significance for the restoration and sustainable development of grassland ecosystems. By offering a novel perspective on alpine grassland management, our results underscore the importance of considering soil fungal communities in conservation strategies to enhance ecosystem functions and services.

## Data Availability

The data presented in the study are deposited in the Zenodo repository; the access URL is https://zenodo.org/records/14630662.

## References

[ref1] Abdalla M. Hastings A. Chadwick D. R. Jones D. L. Evans C. D. Jones M. B.. (2018). Critical review of the impacts of grazing intensity on soil organic carbon storage and other soil quality indicators in extensively managed grasslands. Agric. Ecosyst. Environ. 253, 62–81. doi: 10.1016/j.agee.2017.10.023, PMID: 29398743 PMC5727677

[ref2] Ao D. Wang B. R. Wang Y. B. Chen Y. J. Anum R. Feng C. L.. (2024). Grassland degraded patchiness reduces microbial necromass content but increases contribution to soil organic carbon accumulation. Sci. Total Environ. 951:175717. doi: 10.1016/j.scitotenv.2024.175717, PMID: 39197785

[ref3] Barbi F. Martinović T. Odriozola I. Machac A. Moravcová A. Algora C.. (2025). Disentangling drivers behind fungal diversity gradients along altitude and latitude. New Phytol. doi: 10.1111/nph.70012, PMID: 40007180 PMC12138170

[ref4] Bennett J. A. Klironomos J. (2019). Mechanisms of plant-soil feedback: interactions among biotic and abiotic drivers. New Phytol. 222, 91–96. doi: 10.1111/nph.15603, PMID: 30451287

[ref5] Breidenbach A. Schleuss P. M. Liu S. B. Schneider D. Dippold M. A. de la Haye T.. (2022). Microbial functional changes mark irreversible course of Tibetan grassland degradation. Nat. Commun. 13:2681. doi: 10.1038/s41467-022-30047-7 35562338 PMC9106683

[ref6] Cao L. H. Liu H. M. Zhao S. W. (2011). The distribution of soil water contents and bulk density on degraded grassland at Dangxiong. Acta Agrestia Sin. 19, 746–751. doi: 10.11733/j.issn.1007-0435.2011.05.006

[ref7] Chappell A. Webb N. P. Butler H. J. Strong C. L. McTainsh G. H. Leys J. F.. (2013). Soil organic carbon dust emission: an omitted global source of atmospheric CO_2_. Glob. Change Biol. 19, 3238–3244. doi: 10.1111/gcb.12305, PMID: 23897802

[ref8] Che R. X. Wang Y. F. Li K. X. Xu Z. H. Hu J. M. Wang F.. (2019). Degraded patch formation significantly changed microbial community composition in alpine meadow soils. Soil Tillage Res. 195:104426. doi: 10.1016/j.still.2019.104426

[ref9] Chen B. B. Jiao S. Luo S. W. Ma B. B. Qi W. Cao C. D.. (2021). High soil pH enhances the network interactions among bacterial and archaeal microbiota in alpine grasslands of the Tibetan Plateau. Environ. Microbiol. 23, 464–477. doi: 10.1111/1462-2920.15333, PMID: 33215802

[ref10] Chen J. Shi Z. M. Liu S. Zhang M. M. Cao X. W. Chen M.. (2022). Altitudinal variation influences soil fungal community composition and diversity in alpine–gorge region on the eastern Qinghai–Tibetan Plateau. J. Fungi 8:807. doi: 10.3390/jof8080807, PMID: 36012795 PMC9410234

[ref11] Chen W. Q. Wang J. Y. Meng Z. X. Xu R. Chen J. Zhang Y. J.. (2020). Fertility-related interplay between fungal guilds underlies plant richness-productivity relationships in natural grasslands. New Phytol. 226, 1129–1143. doi: 10.1111/nph.16390, PMID: 31863600

[ref12] Cho N. S. Wilkolazaka A. J. Staszczak M. Cho H. Y. Ohga S. (2009). The role of laccase from white rot fungi to stress conditions. J. Fac. Agric. Kyushu Univ. 54, 81–83. doi: 10.5109/14041

[ref13] Coyte K. Z. Schluter J. Foster K. R. (2015). The ecology of the microbiome: networks, competition, and stability. Science 350, 663–666. doi: 10.1126/science.aad2602, PMID: 26542567

[ref14] de Araujo A. S. F. Bezerra W. M. dos Santos V. M. Nunes L. A. P. L. de Lyra M. D. C. P. Figueiredo M. D. B.. (2017). Fungal diversity in soils across a gradient of preserved Brazilian Cerrado. J. Microbiol. 55, 273–279. doi: 10.1007/s12275-017-6350-6 28127719

[ref15] de Vries F. T. Griffiths R. I. Bailey M. Craig H. Girlanda M. Gweon H. S.. (2018). Soil bacterial networks are less stable under drought than fungal networks. Nat. Commun. 9:3033. doi: 10.1038/s41467-018-05516-7, PMID: 30072764 PMC6072794

[ref16] Faust K. Raes J. (2012). Microbial interactions: from networks to models. Nat. Rev. Microbiol. 10, 538–550. doi: 10.1038/nrmicro2832, PMID: 22796884

[ref17] Hou Y. H. He K. Y. Chen Y. Zhao J. X. Hu H. F. Zhu B. (2021). Changes of soil organic matter stability along altitudinal gradients in Tibetan alpine grassland. Plant Soil 458, 21–40. doi: 10.1007/s11104-019-04351-z

[ref19] Hussain S. Liu H. Liu S. L. Yin Y. F. Yuan Z. Y. Zhao Y. G.. (2022). Distribution and assembly processes of soil fungal communities along an altitudinal gradient in Tibetan Plateau. J. Fungi 7:1082. doi: 10.3390/jof7121082 PMC8706254 34947064

[ref20] Jarvis S. G. Woodward S. Taylor A. F. S. (2015). Strong altitudinal partitioning in the distributions of ectomycorrhizal fungi along a short (300 m) elevation gradient. New Phytol. 206, 1145–1155. doi: 10.1111/nph.13315, PMID: 25655082

[ref21] Ji L. Shen F. Y. Liu Y. Yang Y. C. Wang J. Purahong W.. (2022). Contrasting altitudinal patterns and co-occurrence networks of soil bacterial and fungal communities along soil depths in the cold-temperate montane forests of China. Catena 209:105844. doi: 10.1016/j.catena.2021.105844

[ref22] Jiang M. F. Liu J. Y. Sun H. R. Chen Q. B. Jin H. Yang J. Y.. (2024). Soil microbial diversity and composition response to degradation of the alpine meadow in the southeastern Qinghai-Tibet Plateau. Environ. Sci. Pollut. Res. 31, 26076–26088. doi: 10.1007/s11356-024-32536-2, PMID: 38491240

[ref23] Kang L. Y. Chen L. Y. Zhang D. Y. Peng Y. F. Song Y. T. Kou D.. (2022). Stochastic processes regulate belowground community assembly in alpine grasslands on the Tibetan Plateau. Environ. Microbiol. 24, 179–194. doi: 10.1111/1462-2920.15827, PMID: 34750948

[ref24] Kubartová A. Ranger J. Berthelin J. Beguiristain T. (2009). Diversity and decomposing ability of saprophytic fungi from temperate forest litter. Microb. Ecol. 58, 98–107. doi: 10.1007/s00248-008-9458-8, PMID: 18982382

[ref25] Li C. L. Cao Z. Y. Chang J. J. Zhang Y. Zhu G. L. Zong N.. (2017). Elevational gradient affect functional fractions of soil organic carbon and aggregates stability in a Tibetan alpine meadow. Catena 156, 139–148. doi: 10.1016/j.catena.2017.04.007

[ref26] Li Q. He G. X. Wen T. Zhang D. G. Liu X. N. (2022). Distribution pattern of soil fungi community diversity in alpine meadow in Qilian Mountains of Eastern Qinghai-Tibetan Plateau. Ecol. Indic. 141:109054. doi: 10.1016/j.ecolind.2022.109054

[ref27] Li Y. M. Wang S. P. Jiang L. L. Zhang L. R. Cui S. J. Meng F. D.. (2016). Changes of soil microbial community under different degraded gradients of alpine meadow. Agric. Ecosyst. Environ. 222, 213–222. doi: 10.1016/j.agee.2016.02.020

[ref28] Li C. M. Zhang D. R. Xu G. C. Yan R. Huang Y. Feng L. Q.. (2023). Effects of alpine grassland degradation on soil microbial communities in Qilian Mountains of China. J. Soil Sci. Plant Nutr. 23, 912–923. doi: 10.1007/s42729-022-01092-4

[ref29] Liu D. Liu G. H. Chen L. Wang G. T. Zhang L. M. (2018). Soil pH determines fungal diversity along an elevation gradient in southwestern China. Sci. China Life Sci. 61, 718–726. doi: 10.1007/s11427-017-9200-1 29307110

[ref30] Liu Y. Wang D. Cui Z. Li S. X. Li R. J. Rodrigo-Comino J.. (2023). Alpine meadow patches unevenly regulate runoff and sediment yield generation on the Qinghai-Tibetan Plateau. J. Hydrol. 623:129848. doi: 10.1016/j.jhydrol.2023.129848

[ref31] Liu Y. H. Yang Y. W. Zhang Y. (2018). Redundancy analysis of the relationship between plant functional groups and soil factors in the degraded alpine meadow. J. Ecol. Rural Environ. 34, 1112–1121. doi: 10.11934/j.issn.1673-4831.2018.12.008

[ref32] Liu Y. Zhao L. Liu Y. Huang Z. Shi J. Wang Y.. (2022). Restoration of a hillslope grassland with an ecological grass species (*Elymus tangutorum*) favors rainfall interception and water infiltration and reduces soil loss on the Qinghai-Tibetan Plateau. Catena 219:106632. doi: 10.1016/j.catena.2022.106632

[ref33] Luo Y. Y. Meng Q. T. Zhang J. H. Zhao X. Y. Qin Y. (2014). Species diversity and biomass in relation to soil properties of alpine meadows in the eastern Tibetan Plateau in different degradation stages. J. Glaciol. Geocryol. 36, 1298–1305. doi: 10.7522/j.issn.1000-0240.2014.0155

[ref34] Ma Y. Wang X. L. Ma Y. S. Zhang D. G. (2024). Effects of the degree of alpine meadow degradation on the rhizosphere soil fungal community and the ecological network of dominant species. Acta Pratacult. Sin. 33, 125–137. doi: 10.11686/cyxb2023106

[ref35] Ma B. Wang Y. L. Ye S. D. Liu S. Stirling E. Gilbert J. A.. (2020). Earth microbial co-occurrence network reveals interconnection pattern across microbiomes. Microbiome 8:82. doi: 10.1186/s40168-020-00857-2, PMID: 32498714 PMC7273686

[ref36] Mayer M. Rewald B. Matthews B. Sandén H. Rosinger C. Katzensteiner K.. (2021). Soil fertility relates to fungal-mediated decomposition and organic matter turnover in a temperate mountain forest. New Phytol. 231, 777–790. doi: 10.1111/nph.17421, PMID: 34013982 PMC7611052

[ref37] Miyamoto Y. Nakano T. Hattori M. Nara K. (2014). The mid-domain effect in ectomycorrhizal fungi: range overlap along an elevation gradient on Mount Fuji, Japan. ISME J. 8, 1739–1746. doi: 10.1038/ismej.2014.34, PMID: 24621523 PMC4817612

[ref38] Ning Q. Chen L. Li F. Zhang C. Z. Ma D. H. Cai Z. J.. (2022). Effects of *Mortierella* on nutrient availability and straw decomposition in soil. Acta Pedol. Sin. 59, 206–217. doi: 10.11766/trxb202006020213

[ref39] Njeru C. M. Ekesi S. Mohamed S. A. Kinyamario J. I. Kiboi S. Maeda E. E. (2017). Assessing stock and thresholds detection of soil organic carbon and nitrogen along an altitude gradient in an East Africa mountain ecosystem. Geoderma Reg. 10, 29–38. doi: 10.1016/j.geodrs.2017.04.002

[ref40] Nottingham A. T. Fierer N. Turner B. L. Whitaker J. Ostle N. J. McNamara N. P.. (2018). Microbes follow Humboldt: temperature drives plant and soil microbial diversity patterns from the Amazon to the Andes. Ecology 99, 2455–2466. doi: 10.1002/ecy.2482, PMID: 30076592 PMC6850070

[ref41] Peay K. G. Kennedy P. G. Talbot J. M. (2016). Dimensions of biodiversity in the earth mycobiome. Nat. Rev. Microbiol. 14, 434–447. doi: 10.1038/nrmicro.2016.59, PMID: 27296482

[ref42] Qian J. X. Wang D. Zhao L. R. Cui Z. Li S. X. Liu Y. (2024). Severe degradation and artificial restoration diversely drive runoff and sediment processes in alpine meadows. Geoderma 443:116828. doi: 10.1016/j.geoderma.2024.116828

[ref44] Santolini M. Barabási A. L. (2018). Predicting perturbation patterns from the topology of biological networks. Proc. Natl. Acad. Sci. U.S.A. 115, E6375–E6383. doi: 10.1073/pnas.1720589115, PMID: 29925605 PMC6142275

[ref45] She Y. D. Zhang Z. H. Ma L. Xu W. H. Huang X. Zhou H. K. (2022). Vegetation attributes and soil properties of alpine grassland in different degradation stages on the Qinghai-Tibet Plateau, China: a meta-analysis. Arab. J. Geosci. 15:193. doi: 10.1007/s12517-021-09400-5

[ref46] Siles J. A. Margesin R. (2016). Abundance and diversity of bacterial, archaeal, and fungal communities along an altitudinal gradient in alpine Forest soils: what are the driving factors? Microb. Ecol. 72, 207–220. doi: 10.1007/s00248-016-0748-2, PMID: 26961712 PMC4902835

[ref47] Sun H. R. Liu J. Y. Wu J. H. Hu H. Y. Chen Q. B. Fang H. Y.. (2024). Effects of alpine grassland degradation on soil microbial community structure and metabolic activity in the Qinghai-Tibet Plateau. Appl. Soil Ecol. 200:105458. doi: 10.1016/j.apsoil.2024.105458

[ref48] Tedersoo L. Bahram M. Polme S. Koljalg U. Yorou N. S. Wijesundera R.. (2014). Global diversity and geography of soil fungi. Science 346:1078. doi: 10.1126/science.1256688 25430773

[ref49] Truong C. Gabbarini L. A. Corrales A. Mujic A. B. Escobar J. M. Moretto A.. (2019). Ectomycorrhizal fungi and soil enzymes exhibit contrasting patterns along elevation gradients in southern Patagonia. New Phytol. 222, 1936–1950. doi: 10.1111/nph.15714, PMID: 30689219

[ref50] Van Nuland M. Bailey J. Schweitzer J. (2017). Divergent plant-soil feedbacks could alter future elevation ranges and ecosystem dynamics. Nat. Ecol. Evol. 1:150. doi: 10.1038/s41559-017-0150 28812635

[ref51] Wang Y. C. Lu G. X. Yu H. Du X. F. He Q. Yao S. T.. (2021). Meadow degradation increases spatial turnover rates of the fungal community through both niche selection and dispersal limitation. Sci. Total Environ. 798:149362. doi: 10.1016/j.scitotenv.2021.149362, PMID: 34375268

[ref52] Wang X. S. Michalet R. He S. Wang X. T. (2022). The subalpine shrub *Dasiphora fruticose* alters seasonal and elevational effects on soil microbial diversity and ecosystem functions on the Tibetan Plateau. J. Appl. Ecol. 60, 52–63. doi: 10.1111/1365-2664.14316

[ref53] Wang Q. Wu X. Liu B. Wan J. Jin H. Tao K.. (2022). Deciphering environmental factors driving soil microbial elevational distribution in the southeastern Qinghai-Tibetan Plateau. Eur. J. Soil Biol. 113:103444. doi: 10.1016/j.ejsobi.2022.103444

[ref54] Wardle D. A. Jonsson M. Bansal S. Bardgett R. D. Gundale M. J. Metcalfe D. B. (2012). Linking vegetation change, carbon sequestration and biodiversity: insights from island ecosystems in a long-term natural experiment. J. Ecol. 100, 16–30. doi: 10.1111/j.1365-2745.2011.01907.x

[ref55] Wu Y. P. Liang A. S. Ding M. J. Zhang H. Xu H. Zhang Y. J. (2024). Meadow degradation reduces microbial β diversity and network complexity while enhancing network stability. Appl. Soil Ecol. 204:105733. doi: 10.1016/j.apsoil.2024.105733

[ref56] Wu G. L. Ren G. H. Dong Q. M. Shi J. J. Wang Y. L. (2014). Above- and belowground response along degradation gradient in an alpine grassland of the Qinghai-Tibetan Plateau. Clean Soil Air Water 42, 319–323. doi: 10.1002/clen.201200084

[ref57] Wu X. F. Yang J. J. Ruan H. Wang S. N. Yang Y. R. Naeem I.. (2021). The diversity and co-occurrence network of soil bacterial and fungal communities and their implications for a new indicator of grassland degradation. Ecol. Indic. 129:107989. doi: 10.1016/j.ecolind.2021.107989

[ref58] Xu M. Li X. L. Cai X. B. Gai J. P. Li X. L. Christie P.. (2014). Soil microbial community structure and activity along a montane elevational gradient on the Tibetan Plateau. Eur. J. Soil Biol. 64, 6–14. doi: 10.1016/j.ejsobi.2014.06.002

[ref59] Yang Y. F. Gao Y. Wang S. P. Xu D. P. Yu H. Wu L. W.. (2014). The microbial gene diversity along an elevation gradient of the Tibetan grassland. ISME J. 8, 430–440. doi: 10.1038/ismej.2013.146, PMID: 23985745 PMC3906809

[ref60] Yang N. Li X. X. Liu D. Zhang Y. Chen Y. H. Wang B.. (2022). Diversity patterns and drivers of soil bacterial and fungal communities along elevational gradients in the Southern Himalayas, China. Appl. Soil Ecol. 178:104563. doi: 10.1016/j.apsoil.2022.104563

[ref61] Yang Y. Shi Y. Kerfahi D. Ogwu M. C. Wang J. J. Dong K.. (2021). Elevation-related climate trends dominate fungal co-occurrence network structure and the abundance of keystone taxa on Mt. Norikura, Japan. Sci. Total Environ. 799:149368. doi: 10.1016/j.scitotenv.2021.149368, PMID: 34352461

[ref62] Yu Z. Lee C. B. Kerfahi D. Li N. Yamamoto N. Yang T.. (2024). Elevational dynamics in soil microbial co-occurrence: disentangling biotic and abiotic influences on bacterial and fungal networks on Mt. Seorak. Soil Ecol. Lett. 6, 240–246. doi: 10.1007/s42832-024-0246-2

[ref63] Zhang F. Li Y. M. Ji B. M. Dong S. K. (2024). Spatial distribution and drivers of arbuscular mycorrhizal fungi on the Tibetan Plateau. Front. Plant Sci. 15:1427850. doi: 10.3389/fpls.2024.1427850, PMID: 39045593 PMC11264307

[ref64] Zhang W. J. Xue X. Peng F. You Q. G. Hao A. H. (2019). Meta-analysis of the effects of grassland degradation on plant and soil properties in the alpine meadows of the Qinghai-Tibetan Plateau. Glob. Ecol. Conserv. 20:e00774. doi: 10.1016/j.gecco.2019.e00774

[ref65] Zhao X. Song Y. Q. Xu T. T. Xu M. Cai J. T. Wang L.. (2021). Edge effects and spatial degradation process in highly fragmented grassland—impact on soil microbial community. Ecol. Indic. 132:108307. doi: 10.1016/j.ecolind.2021.108307

[ref66] Zhou H. Zhang D. G. Jiang Z. H. Sun P. Xiao H. L. Wu Y. X.. (2019). Changes in the soil microbial communities of alpine steppe at Qinghai-Tibetan Plateau under different degradation levels. Sci. Total Environ. 651, 2281–2291. doi: 10.1016/j.scitotenv.2018.09.336 30326458

